# Effects of Hydraulic Retention Time on Removal of Cr (VI) and p-Chlorophenol and Electricity Generation in *L. hexandra*-Planted Constructed Wetland–Microbial Fuel Cell

**DOI:** 10.3390/molecules29194773

**Published:** 2024-10-09

**Authors:** Tangming Li, Peiwen Yang, Jun Yan, Mouyixing Chen, Shengxiong You, Jiahuan Bai, Guo Yu, Habib Ullah, Jihuan Chen, Hua Lin

**Affiliations:** 1 Guangxi Key Laboratory of Theory and Technology for Environmental Pollution Control, Guilin University of Technology, Guilin 541006, China; litangming@glut.edu.cn (T.L.); aohandeid@gmail.com (P.Y.); yanjun@glut.edu.cn (J.Y.); chenmouyixing@glut.edu.cn (M.C.); 18585228219@163.com (S.Y.); 2120210512@glut.edu.cn (J.B.); yuguo@glut.edu.cn (G.Y.); xiaonianhua2023@glut.edu.cn (J.C.); 2 Innovation Center of Yangtze River Delta, Zhejiang University, Hangzhou 311400, China; habib901@mail.ustc.edu.cn; 3 Collaborative Innovation Center for Water Pollution Control and Water Safety in Karst Areas, Guilin University of Technology, Guilin 541000, China

**Keywords:** constructed wetland–microbial fuel cell, configurations, wastewater purification, electricity generation, *Leersia hexandra* Swartz, hydraulic retention time

## Abstract

Hexavalent chromium (Cr (VI)) and para-chlorophenol (4-CP) are prevalent industrial wastewater contaminants that are recalcitrant to natural degradation and prone to migration in aquatic systems, thereby harming biological health and destabilizing ecosystems. Consequently, their removal is imperative. Compared to conventional chemical treatment methods, CW-MFC technology offers broader application potential. *Leersia hexandra* Swartz can enhance Cr (VI) and 4-CP absorption, thereby improving wastewater purification and electricity generation in CW-MFC systems. In this study, three CW-MFC reactors were designed with *L. hexandra* Swartz in distinct configurations, namely, stacked, multistage, and modular, to optimize the removal of Cr (VI) and 4-CP. By evaluating wastewater purification, electrochemical performance, and plant growth, the optimal influent hydraulic retention time (HRT) was determined. The results indicated that the modular configuration at an HRT of 5 days achieved superior removal rates and power generation. The modular configuration also supported the best growth of *L. hexandra*, with optimal photosynthetic parameters, and physiological and biochemical responses. These results underscore the potential of modular CW-MFC technology for effective detoxification of complex wastewater mixtures while concurrently generating electricity. Further research could significantly advance wastewater treatment and sustainable energy production, addressing water pollution, restoring aquatic ecosystems, and mitigating the hazards posed by Cr (VI) and 4-CP to water and human health.

## 1. Introduction

The printing, dyeing, and leather industries worldwide generate industrial wastewater laden with hazardous pollutants such as hexavalent chromium (Cr (VI)) and p-chlorophenol (4-CP) [1]. In China, approximately 3906 kg of Cr (VI) and 14,000 tons of 4-CP are released into the environment annually [2,3]. Cr (VI) poses severe health risks, including respiratory, dermal, and tissue damage, with high carcinogenic potential and prolonged latency [4]. 4-CP, a persistent organic pollutant, exhibits environmental persistence, bioaccumulation, high mobility, and toxicity, potentially causing neurological symptoms upon prolonged exposure through contaminated drinking water [5,6]. Numerous studies have demonstrated that Cr (VI) and 4-CP pose significant threats to the ecological environment and human health, exhibiting carcinogenic, teratogenic, and mutagenic properties. Therefore, efficient treatment of industrial wastewater is imperative [4,7]. Conventional methods like electrochemical treatment, membrane separation, and Fenton advanced oxidation face limitations in scalability due to their complexity and cost [8,9,10,11,12]. Therefore, the search for a sustainable and efficient wastewater treatment process is particularly essential. Constructed wetland emerges as a promising green technology offering ecological benefits such as low investment, energy efficiency, and environmental protection. The earliest constructed wetland, established in Germany, utilized *Phragmites australis* [13]. With advancements in wetland technology, a variety of wetland plants have since been employed. Notably, *Leersia hexandra*, *Medicago sativa* L., and *Vicia sativa* L. have proven effective in the removal of phenolic compounds [14]. *Leersia hexandra* Swartz, identified as the first wet-growing graminaceous chromium hyperaccumulator in China [15], is characterized by its perennial nature, high reproductive capacity, large biomass, rapid growth, and tolerance to 4-CP [8,16]. Thus, *L. hexandra* is an ideal candidate for constructed wetlands to remediate chromium and 4-CP from industrial wastewater [8,17]. Wu et al. [18] utilized *Leersia hexandra* Swartz for the removal of Cr (VI) in constructed wetlands, discovering that the plant increased organic matter content in the wetland matrix, thereby enhancing the constructed wetland’s purification capacity for Cr (VI). Additionally, *Leersia hexandra* Swartz has been effectively applied to the treatment of electroplating wastewater [19].

However, constructed wetlands have significant limitations, including susceptibility to seasonal temperature fluctuations, low resistance to pollution load, and a propensity for clogging [20]. Liu et al. [17] found that further improvement of chromium removal in constructed wetlands was limited by the inefficiency of Cr (VI) reduction to Cr (III) in *L. hexandra*-planted constructed wetlands due to a lack of electron donors. In recent years, constructed wetland–microbial fuel cells (CW-MFCs) have emerged as a promising technology for wastewater treatment and electricity generation, compared to standalone CW or MFC systems, offering superior purification of both organic and heavy metal contaminants, and significant promise for future applications [21,22].

The CW-MFC technology operates on the principle of electrochemically active bacteria (EABs) degrading pollutants in the anodic area, leading to the generation of electrons and protons; while electrons are conveyed to the cathode through external circuits, the protons arrive at the cathode by diffusion from the anodic area. Natural redox conditions exist within the system, and the electrical energy generated by MFCs can potentially enhance the removal rates of CWs [23,24]. For instance, Mu et al. [25] achieved a 99% removal rate of Cr (VI) and maximized power density using volcanic rock as filler in CW-MFCs. Similarly, Wang et al. [8] demonstrated that an *L. hexandra*-planted CW-MFC exhibited the highest electricity generation and detoxification capacity for Cr (VI) and 4-CP effluents. Shi et al. [26] proposed that *L. hexandra* fulfills dual functions in CW-MFC systems: it facilitates pollutant uptake (e.g., heavy metals, phenolic compounds) and creates an optimal root environment for microorganisms. This symbiosis indirectly augments the power generation efficiency of CW-MFC by promoting pollutant degradation or reduction through microbial activity and enhancing the metabolism of electrochemically active bacteria (EABs).

To enhance pollutant removal efficiency and address low electrochemical performance, various CW-MFC configurations have been developed, including stacked [27,28], multistage [29,30], and modular configurations [31]. The stacked configuration, characterized by its vertical arrangement and compact footprint, efficiently reduces land usage and optimizes space utilization [32]. Tamta et al. [32] employed this configuration for high-load domestic wastewater treatment, achieving COD and NH_4_^+^-N removal rates of 98.5% and 90.4%, respectively. However, conventional stacked configurations suffer from partial polarization, impairing power production efficiency. Muhammad et al. [33] addressed this issue by optimizing the configuration, attaining a maximum power density of 9.02 mW·m^2^. The multistage configuration, involving CW-MFC reactors connected in series with varying flow regimes, enhances the removal of pollutants such as organic matter, nitrogen, and phosphorus while improving power generation compared to single-chamber CW-MFCs [34]. Gupta et al. [35] developed a multistage CW-MFC system that achieved simultaneous nitrification–denitrification processes, with COD, NH_4_^+^-N, and total phosphorus removal rates of 96.37%, 85.14%, and 96.03%, respectively. This configuration also demonstrates superior efficacy for heavy metal–polluted wastewater [35]. The modular configuration, characterized by its scalability to meet actual wastewater treatment demands, facilitates the partitioning, classification, and hierarchical treatment of wastewater for improved outcomes [36,37]. Tamta et al. [32] utilized a modular CW-MFC reactor for high-load domestic wastewater treatment, achieving COD and NH_4_^+^-N removal rates of 98.5% and 90.4%, respectively. Additionally, Liu et al. [38] employed a dual–anode modular CW-MFC system for ammonia treatment under low-temperature conditions, increasing average ammonia removal efficiency by 18.1%.

In conclusion, CW-MFC has great potential for treating wastewater in the future [39]. These configurations have been shown to significantly improve the removal rates of Cr (VI) and 4-CP [8]. However, the effectiveness of CW-MFCs in purifying co-polluted wastewater and electricity generation is influenced by several factors, with the hydraulic retention time (HRT) being particularly critical [27,40,41]. The HRT profoundly impacts system stability, power production efficiency, and water purification performance [42,43]. An insufficient HRT limits microbial contact with pollutants, compromising both pollutant removal and power generation [23]. For example, Zhang et al. [44] observed a notable decline in power production voltage when the HRT was reduced from 18 to 12 h. This phenomenon may be attributed to the accelerated flow rate of the short HRT, which deprives electrochemically active bacteria of essential nutrients required for normal proliferation and electricity generation. Moreover, incomplete consumption of dissolved oxygen in the anode area inhibits the activity of these bacteria. Conversely, Li et al. [45] found that extending the HRT to 16 h increased the removal rates of ibuprofen by 14.6%, bisphenol A by 23.7%, and COD by 20.87%. Analysis of the microbial community structure reveals that extending the HRT alters the microbial composition and potentially adjusts the pollutant volumetric loading, thereby enhancing pollutant removal rates [40]. Nonetheless, excessively long HRTs have been associated with diminished water purification and power production, as reported by Minakshi et al. [46] and Fang et al. [21]. An excessively long HRT fails to supply adequate carbon sources for EABs, thereby impairing the system’s electricity generation performance [47]. Furthermore, an extended HRT increases both the time and operational costs of wastewater treatment [48]. Thus, optimizing the HRT requires balancing treatment efficacy and cost. Numerous studies suggest that an optimal HRT of 3 d could achieve this balance [21,48,49].

This study aimed to investigate the impact of varying HRT on purification efficiency, electrochemical performance, and physiological and biochemical responses of *L. hexandra* across three CW-MFC configurations, thereby providing a theoretical basis for the application of CW-MFCs in treating Cr (VI) and 4-CP co-polluted wastewater while simultaneously generating electricity.

The nomenclatures are shown in Table 1.

## 2. Results and Discussion

### 2.1. The Effect of the HRT on Wastewater Purification Performance with Different Configurations

**Figure 1:**
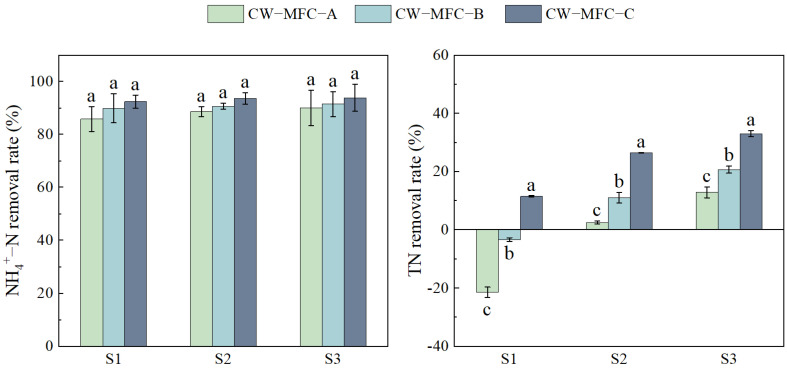
NH_4_^+^−N and TN contents in the effluent of different CW-MFC system configurations. Different lowercase letters indicate significant differences between the treatments (*p* < 0.05).

Figure 1 illustrates the enhanced removal rates of NH₄⁺-N and TN with an extended HRT. NH₄⁺-N removal rates showed no significant difference and TN removal rates showed significant differences: the increase in CW-MFC-A, CW-MFC-B, and CW-MFC-C was 20.42%, 13.80%, and 16.95%, respectively, for TN. Compared to CW-MFC-B and CW-MFC-C, CW-MFC-A showed the greatest improvement in TN removal rates, while CW-MFC-C demonstrated the highest overall removal rates. The NH_4_^+^-N in effluent reached first-level standard A (<5 mg·L^−1^), and the TN in effluent reached first-level standard B (<20 mg·L^−1^).

**Figure 2:**
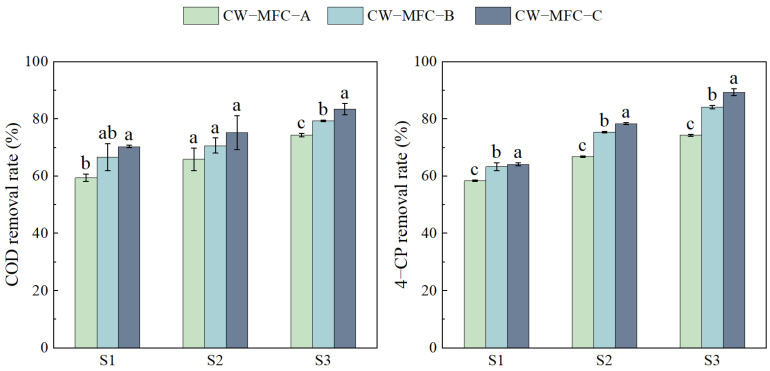
Removal rates of COD and 4-CP in the effluent of different CW-MFC system configurations. Different lowercase letters indicate significant differences between the treatments (*p* < 0.05).

Figure 2 shows a rising trend in the removal efficiencies of COD and 4-CP with an extended HRT. COD removal rates and TN removal rates showed significant differences. From S2 to S3, all three CW-MFC configurations showed the best improvements in COD removal: the increase in CW-MFC-A, CW-MFC-B, and CW-MFC-C was 8.53%, 8.63%, and 8.25%, respectively. From S1 to S2, the configurations demonstrated notable improvements in 4-CP removal: the increase of CW-MFC-A, CW-MFC-B, and CW-MFC-C was 8.47%, 12.04%, and 14.27%, respectively. While CW-MFC-A showed the best improvement in COD removal compared to CW-MFC-B and CW-MFC-C, CW-MFC-C achieved the highest overall removal rates for both COD and 4-CP. The COD in effluent reached first-level standard A (<50 mg·L^−1^), and the 4-CP in effluent did not reach the standard value (<0.6 mg·L^−1^).

**Figure 3:**
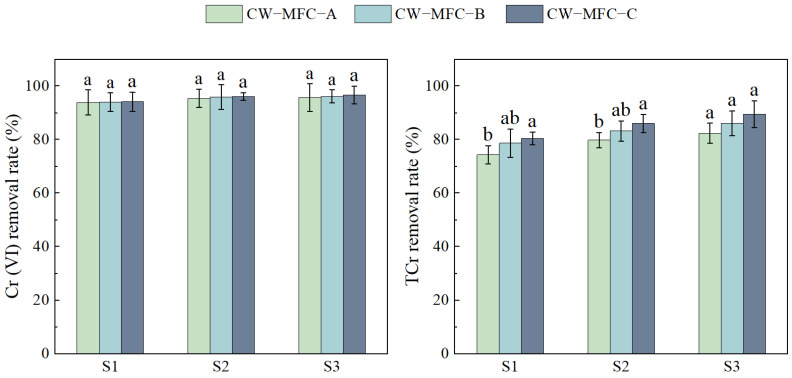
Removal rates of Cr (Ⅵ) and TCr in the effluent of different CW−MFC system configurations. Different lowercase letters indicate significant differences between the treatments (*p* < 0.05).

Figure 3 shows the enhanced removal rates of Cr (VI) and total chromium (TCr) with an extended HRT. However, Cr (VI) removal rates and TCr removal rates showed no significant differences. Compared to CW-MFC-A and CW-MFC-B, CW-MFC-C exhibited the highest removal efficiency for Cr. The Cr (VI) in effluent did not reach the standard value (<0.05 mg·L^−1^), and the TCr in effluent did not reach the standard value (<0.1 mg·L^−1^).

During S3, all three CW-MFC reactor configurations exhibited optimal purification of NH_4_^+^-N, TN, COD, 4-CP, Cr (VI), and TCr, confirming that an extended HRT enhances the wastewater treatment efficiency of CW-MFC systems, as corroborated by Jiang et al. [50]. The primary mechanism for pollutant removal in CW-MFC systems is microbial degradation [51]. A short HRT reduces contact time between microorganisms and organic substrates, diminishing microbial activity and thereby the efficacy of pollutant removal [52]. Conversely, extending the HRT allows for sufficient microbial interaction with substrates, promoting growth and reproduction, which enhances pollutant degradation [52]. However, the improvement in pollutant removal during S3 was less marked compared to S2. This attenuation is likely due to lower organic matter content and increased anoxic conditions following HRT extension, which collectively suppressed microbial activity relative to S2 [53,54]. Moreover, an excessively extended HRT can negatively impact effluent quality and increase treatment costs [23]. Among the three configurations, CW-MFC-C demonstrated superior purification performance. This can be attributed to its robust electricity generation, wherein EABs produce more electrons, facilitating rapid pollutant conversion and degradation [55,56]. When the HRT was extended, CW-MFC-A showed the most significant improvement in pollutant removal compared to CW-MFC-B and CW-MFC-C. This enhancement can be linked to CW-MFC-A’s inside-out, top-to-bottom cascade process (drop aeration process) [27], which effectively increases dissolved oxygen concentration in the reactor, thereby improving overall pollutant removal efficiency.

As previously stated, microbial activity is the primary factor influencing wastewater treatment in CW-MFC. Numerous studies have demonstrated that microbial community structures are modified under the impact of Cr (VI) and 4-CP [14], involving chemical reactions such as Cr (VI) reduction and 4-CP dechlorination and degradation [14,57]. Wang et al. [14] conducted microbial analyses within a *L. hexandra*-planted CW-MFC after the addition of Cr (VI) and 4-CP. They discovered a significant abundance of *Proteobacteria*, *Bacteroidetes*, and *Acidobacterium*, with *Proteobacteria* capable of removing Cr (VI) and 4-CP [25,58]. Both *Bacteroidetes* and *Acidobacterium* harbored heavy metal resistance or reduction genes and exhibited strong tolerance to chlorophenols [59]. At the genus level, *Simplicispira, Cloacibacterium*, *Rhizobium*, and *Rhodopseudomonas* demonstrated high abundance and were implicated in the adsorption, reduction, and removal of Cr (VI) [14], while *Simplicispira*, *Cloacibacterium*, *Rhizobium*, *Paludibacter*, and *Rhodopseudomonas* were involved in the degradation of 4-CP [14,60]. Investigating microbial activity patterns is crucial for comprehensively understanding the water purification mechanisms of CW-MFC and enhancing its performance. Therefore, further exploration of microbial activities in subsequent experiments is essential.

### 2.2. The Effect of the HRT on Electrochemical Performance with Different Configurations

**Figure 4:**
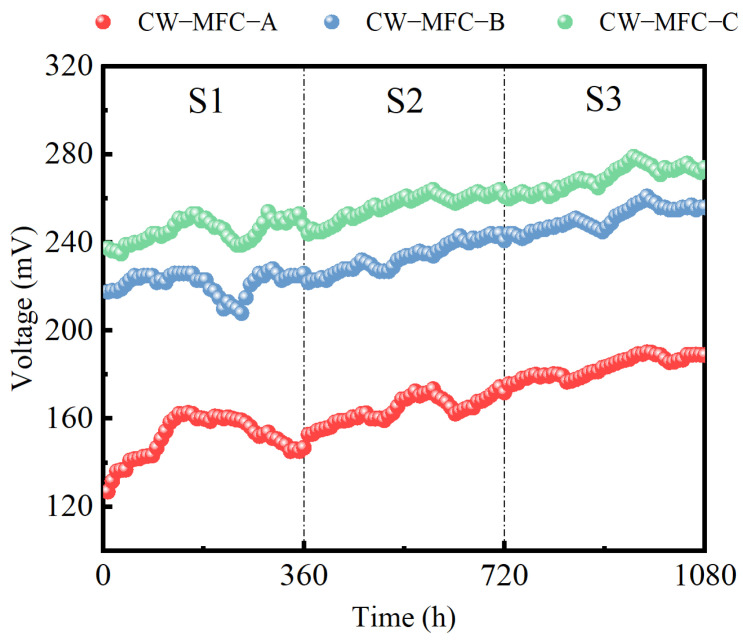
Voltage of different CW-MFC system configurations.

Figure 4 illustrates the recovery and rise in output voltages of the three CW-MFC configurations with an extended HRT, albeit with fluctuations. During S3, compared to S1, the peak output voltages reached 189.4, 261.0, and 279.1 mV, representing increases of 16.2%, 14.4%, and 9.9%. From S2 to S3, the maximum output voltage of CW-MFC-A, CW-MFC-B, and CW-MFC-C was improved by 8.54%, 6.97%, and 5.72%, respectively. While CW-MFC-A showed the greatest enhancement in maximum output voltage, CW-MFC-C demonstrated the best overall electricity generation performance. When the HRT is short, the rapid water current swiftly removes organic matter essential for EABs, hindering their normal reproduction and electricity generation. Furthermore, some organic matter is not completely degraded and reaches the cathode, fostering microbial growth that competes in the O_2_ reduction reaction, thereby diminishing the electricity generation performance. Additionally, the incomplete consumption of dissolved oxygen in the anode region inhibits bacterial activity to some extent [61,62]. As the HRT increases, the contact time between EABs and organic matter lengthens, facilitating greater electron generation from organic matter degradation and thereby enhancing the system’s voltage output [63].

**Figure 5:**
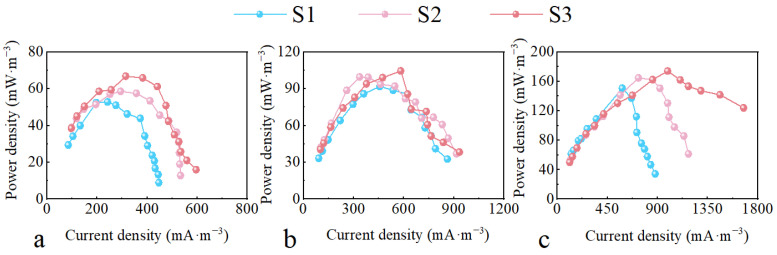
Power density curve of different CW-MFC system configurations. (**a**) CW-MFC-A; (**b**) CW-MFC-B; (**c**) CW-MFC-C.

Figure 5 shows the increasing trend in power densities of CW-MFC reactors as the HRT was extended from 3 to 5 days. Notably, S3 achieved the highest maximum power densities: CW-MFC-A, CW-MFC-B, and CW-MFC-C were recorded at 66.77, 104.5, and 174.0 mW·m^−3^, respectively. Compared to S1, these maximum power densities were enhanced by 26.46%, 13.81%, and 15.53%. Extending the HRT from 3 to 4 d improved CW-MFC-A, CW-MFC-B, and CW-MFC-C maximum power densities by 9.71%, 8.43%, and 9.33%, respectively, while extending from 4 to 5 days resulted in increases of 12.4%, 5.14%, and 5.38%.

**Figure 6:**
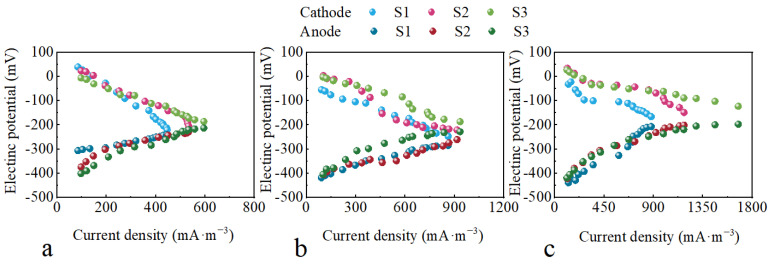
Cathode and anode potential of different CW-MFC system configurations. (**a**) CW-MFC-A; (**b**) CW-MFC-B; (**c**) CW-MFC-C.

Two key observations were made regarding the power production performance of CW-MFCs. Firstly, CW-MFC-C demonstrated superior power generation performance, evidenced by its larger maximum power density and current density interval ranges compared to CW-MFC-A and CW-MFC-B [8]. Secondly, CW-MFC-A exhibited the most significant increase in maximum power density compared to CW-MFC-B and CW-MFC-C. As shown in Figure 6a, extending the HRT effectively reduced the anode potential of CW-MFC-A, thereby increasing the cathode potential difference and ultimately enhancing its power density [8].

The analysis indicated that extending the HRT enhances electricity generation performance. This improvement is attributed to the gradual deceleration of wastewater flow in the CW-MFC, allowing nutrients to be fully utilized by domesticated electrochemically active bacteria. This promotes bacterial growth and reproduction, enhances wastewater purification, and increases electron generation, thereby improving electron transport capacity, reducing internal resistance, and boosting power density [8]. Among the three configurations, CW-MFC-C exhibited the highest electricity generation performance during S3, attributed to its superior wastewater purification, optimal anodic performance, and stable anodic biofilm [64]. Conversely, CW-MFC-A showed the most enhancement in electricity generation production, likely due to its unique inside-out, top-to-bottom cascade process (drop aeration process) [27]. This process effectively increases dissolved oxygen concentration, enabling the air cathode of CW-MFC-A to accept more electron acceptors, thereby significantly enhancing its electricity generation performance. Compared with other studies (Table 2), our study demonstrates stronger electricity generation and higher detoxification of polluted water containing phenolic compounds and heavy metals.

As previously noted, the electricity generation performance of CW-MFCs is intrinsically linked to microbial activities. Variations in the abundance of EABs significantly influence the power output of these systems [65]. Wang et al. [14] investigated the EABs in CW-MFCs cultivated with *L. hexandra* and discovered that the introduction of Cr (VI) and 4-CP increased the abundance of *Proteobacteria*, *Bacteroidetes*, *Firmicutes*, and *Spirochaetae* at the cathode, anode, and within the root system of L. hexandra. These taxa are typically recognized as major EABs [66]. At the genus level, *Geobacter*, *Acinetobacter*, *Spirochaeta*, and *Bacillus* exhibited higher abundance and were pivotal in CW-MFC electricity generation [14,67]. Intermediates from 4-CP degradation, such as benzoic acid, can function as electron donors [68], and *Bacillus* can directly transfer electrons to the anode and participate in Cr (VI) reduction [69]. Investigating the activity patterns of these microorganisms is essential for a deeper understanding of the electro-production mechanisms in CW-MFCs, facilitating performance enhancements. Therefore, further exploration of microbial activities in subsequent experiments is imperative.

**Table 2:** The electricity generation and pollutants removal performance of recent CW–MFC studies.

HRT	Plant	Pollutant and Removal Rate	Maximum Power Density	Voltage	Reference
10 h	*Lycoris radiata*	Cr (Ⅵ) (99.8%) TCr (99.6%)	23.72 W/m^3^	400 mV	[70]
1.5 d	*L. hexandra*	Cr (Ⅵ) (99%)	64.2 mW/m^2^	500 mV	[71]
2.5 d	*L. hexandra*	Cr (Ⅵ) (98.5%) 4-CP (42.1%)	64.6 mW/m^3^	516 mV	[8]
3 d	*Iris tectorum*	Pb (84.86%)	7.43 mW/m^2^	343 mV	[72]
3 d	N/A	Cr (Ⅵ) (93%) COD (89.2%)	426.8 mW/m^3^	558.1 mV	[73]
4 d	*Acorus calamus* L.	TCr (95.6%) COD (86.5%)	46.63 mW/m^2^	650 mV	[74]
5 d	*L. hexandra*	Cr (Ⅵ) (96.7%) 4-CP (89.3%)	174 mW/m^3^	279.1 mV	Present study
6.5 d	*L. hexandra*	Cr (Ⅵ) (99%) 4-CP (78.6%)	72.25 mW/m^3^	543 mV	[8]

### 2.3. Effects of HRT on Physiological and Biochemical Responses of L. hexandra with Different Configurations

**Figure 7:**
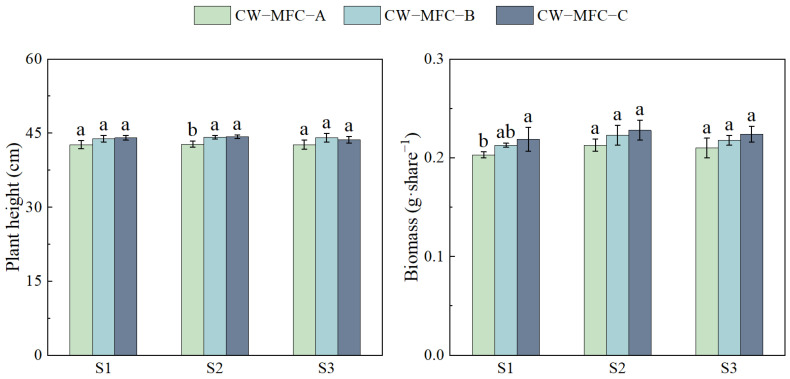
Plant height and biomass of *L. hexandra.* Different lowercase letters indicate significant differences between the treatments (*p* < 0.05).

Figure 7 illustrates the trend in plant height and biomass of *L. hexandra* with an extended HRT. However, there was no significant difference in both plant height and biomass, which may be caused by the better chromium removal effect in all reactors and the lower inhibitory effect of chromium on *L. hexandra* [75]. S2 exhibited the maximum plant height and biomass of *L. hexandra*. Compared to CW-MFC-A and CW-MFC-B, CW-MFC-C had the best overall situation in terms of plant height and biomass.

**Figure 8:**
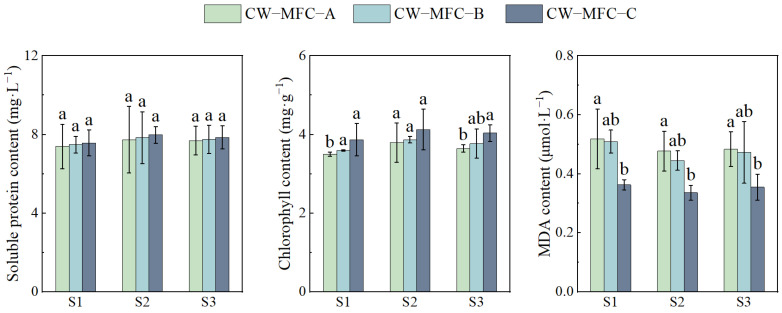
Soluble protein, chlorophyll, and MDA contents in leaves of *L. hexandra.* Different lowercase letters indicate significant differences between the treatments (*p* < 0.05).

Figure 8 shows the trends of soluble protein and chlorophyll content increases, alongside a decrease in MDA content in leaves with an extended HRT in CW-MFC systems. The chlorophyll content and MDA content showed significant differences, and soluble protein content showed no significant difference, which may be caused by the better chromium removal in each reactor and the lower chromium stress on *L. hexandra*. S2 exhibited the most substantial changes, with chlorophyll increasing by 8.60%, 7.80%, and 6.72%, and MDA decreasing by 7.69%, 9.80%, and 8.69%, compared to S1.

**Figure 9:**
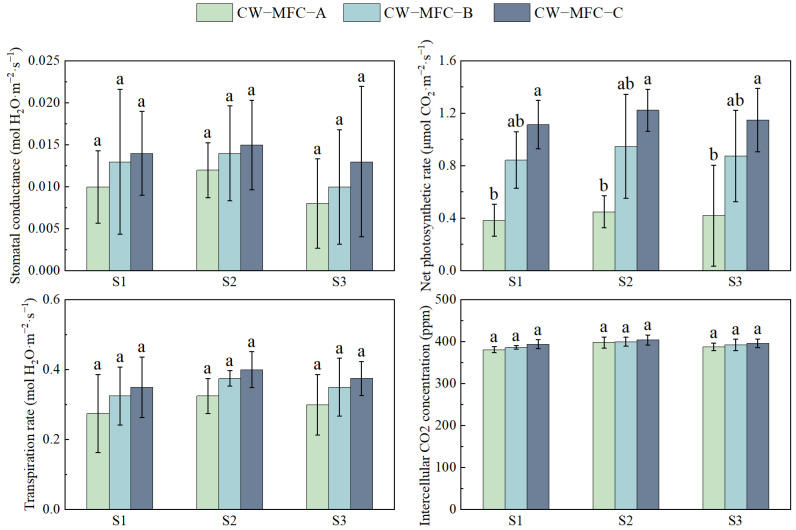
Photosynthesis parameters of leaves of *L. hexandra*. Different lowercase letters indicate significant differences between the treatments (*p* < 0.05).

Figure 9 shows that photosynthesis parameters of *L. hexandra* peaked during S2 across all reactors. The net photosynthetic rate showed significant difference, but stomatal conductance, transpiration rate, and intercellular CO_2_ concentration showed no significant difference, which may be caused by the better removal of pollutants from all the reactors, and *L. hexandra* suffered from a low level of stress in all reactors. Plant photosynthesis is typically constrained by various factors. Some studies have suggested that plant photosynthesis may be predominantly influenced by stomatal limitations in *L. hexandra* [76,77], while the potential influence of non-stomatal factors cannot be disregarded [78]. In the present experiment, photosynthesis is likely influenced by both stomatal and non-stomatal factors.

**Figure 10:**
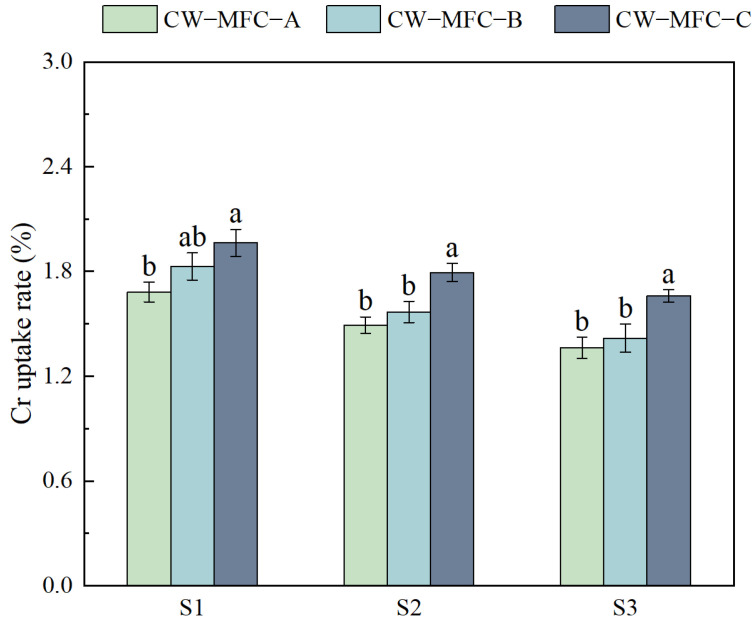
The Cr uptake rate of *L. hexandra*. Different lowercase letters indicate significant differences between the treatments (*p* < 0.05).

Figure 10 illustrates that the Cr uptake rates of three distinct CW-MFC configurations in *L. hexandra* declined with an extended HRT. Cr uptake rates showed significant differences. When the HRT was extended from 3 to 4 d, the Cr uptake rates of the three configurations in *L. hexandra* decreased to 1.49%, 1.56%, and 1.79%. Further extension of the HRT from 4 to 5 d resulted in continued decline to 1.36%, 1.42%, and 1.66%.

The analysis indicates that extending the HRT mitigates the toxicity of 4-CP and Cr (VI) co-pollution to *L. hexandra* while improving the removal of other contaminants in synthetic wastewater. This enhancement is attributable to two primary factors. Firstly, extending the HRT within the 15-day experimental period reduces the total volume of synthetic wastewater treated by each CW-MFC, thereby lessening the toxic burden on *L. hexandra* [8]. Secondly, phytoremediation with *L. hexandra* requires extended duration, and increasing the HRT allows better acclimatization to polluted conditions, indirectly improving pollutant remediation efficacy [79,80]. However, an excessively long HRT can deplete organic substrates, hindering plant growth and adversely affecting wastewater purification and power production [23]. Upon examining the photosynthesis parameters and physiological and biochemical responses of *L. hexandra*, it was observed that S3 exhibited a slight reduction in growth compared to S2. Despite this, S3 showed superior wastewater purification and electricity production among the three CW-MFC configurations, suggesting that a 5-day HRT is optimal. Comparative analysis of the configurations revealed that CW-MFC-C’s *L. hexandra* leaves were least affected by 4-CP and Cr (VI) stress, indicating its superior purification efficacy. Conversely, CW-MFC-A showed more significant changes in photosynthetic and physiological responses with an extended HRT, highlighting greater improvements in wastewater purification.

Additionally, unlike 4-CP, which degrades to CO_2_, heavy metals such as chromium cannot be degraded [14]. Consequently, chromium accumulates in *L. hexandra* and may be re-released into the water upon the plant’s apoptosis. Therefore, it is imperative to regularly harvest and safely dispose of *L. hexandra* once chromium accumulation is complete. Currently, incineration and gasification are the predominant treatment methods, albeit with high costs and potential secondary pollution due to heavy metal release at elevated temperatures [81,82]. Emerging resource-based technologies such as green phytometallurgy and hydrothermal reforming, though still in the developmental stages, have promising future applications [83,84].

## 3. Materials and Methods

### 3.1. System Setup

Granular activated carbon with a particle size of 6–8 mm was used as the electrode filler in the experiment. The cathode used a 6 mm diameter graphite electrode rod, and the anode used a 6 mm diameter copper electrode rod as an electron conductor. Morever, 6 mm diameter copper electrode rods were also used as electron conductors for the electrodes. The skeleton of the electrodes in each reactor was constructed using stainless steel wire mesh. The wire mesh was composed of 304 stainless steel, which has a 5 mesh structure and 0.6 mm diameter, and was cut into stainless steel mesh pieces of corresponding sizes according to the dimensions shown in Figure S1. Stainless steel mesh was used to wrap the electrode packing and electron conductor, and fixed with nylon ties to form a three-dimensional mesh cage electrode. The cathode volumes of stacked, multistage, and modular were 792, 803, and 806 cm^3^, and the anode volumes were 440, 450, and 442 cm^3^.

**Figure 11:**
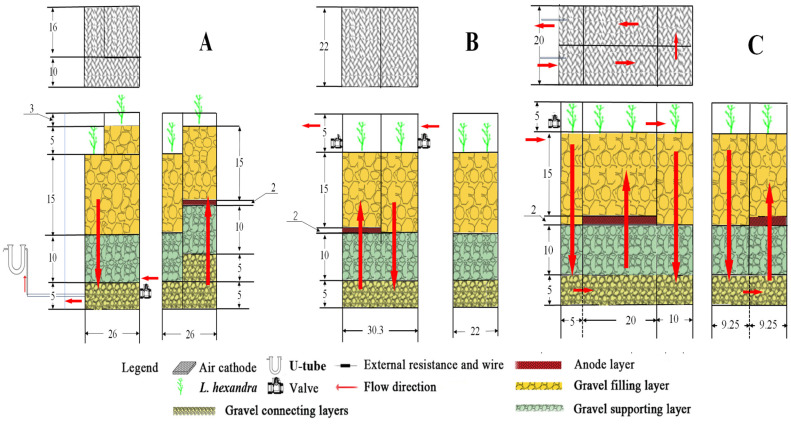
Stereoscopic structure diagram of different CW−MFC system configurations. Note: the units in the figure are cm. (**A**) Stacked configuration; (**B**) multistage configuration; (**C**) modular configuration.

Three different configurations of CW-MFC reactors were constructed using Plexiglas. Each reactor had a body wall of 5 mm thickness, (20.37 ± 0.27) L reactor volume, and (6.12 ± 0.10) L effective volume. The CW-MFC-A, CW-MFC-B, and CW-MFC-C adopt stacked, multistage, and modular configurations, respectively, as shown in Figure 11. Synthetic wastewater flows in each CW-MFC reactor following the direction of the arrows in the figure and eventually overflows from each reactor.

White gravel was used as the filler material in all layers of the CW-MFC reactor, including the connecting, supporting, and filling layers. The connecting gravel layer had a 3–5 cm grain size and 5 cm height. The supporting gravel layer had a 5–6 cm grain size and 10 cm height. The filling gravel layer had a 6–8 cm grain size and 15 cm height. The cathode and anode electrodes of each CW-MFC were spaced 15 cm apart. The cathode, anode, and a 2000 Ω resistor box were connected by titanium wire to achieve a closed loop. The liquid level in the reactor was consistently maintained below the cathode surface to facilitate the formation of a closed loop and air cathode.

### 3.2. Inoculation and Initiation of CW–MFC

The sludge was obtained from the Yanjing Brewery sewage treatment plant (Guilin, China), then anaerobically incubated with a nutrient solution for 7 days, following the formulation described by Liu et al. [85]. The substrate and nutrient solution were mixed at a 1:1 volume ratio to create an inoculated mixture. This mixture was pumped from the bottom of each reactor to fill the lower surface of the cathode using a peristaltic pump. The synthetic wastewater was pumped from the inlet valve using another peristaltic pump to regulate the pH to 7.00 ± 0.10 [86]. After stabilizing for 30 days, the experiment was started once the output voltage stabilized. The details of the synthetic wastewater composition can be found in Table 3.

Three CW-MFC reactor systems were operated continuously at room temperature (25 ± 5 °C), 12 h light duration, and 7000 Lx light intensity in a laboratory environment. The HRT of each reactor was controlled by modifying the flow rate of the peristaltic pumps. Each operating stage was 15 days, and the total stage was 75 days. Table 4 shows the different operating conditions of the CW-MFCs at 150 mg·L^−1^ COD, 150 mg·L^−1^ 4-CP, and 28 mg·L^−1^ ammonia nitrogen (total nitrogen). At the start of each stage, 30 pre-cultured *L. hexandra* plants with similar growth status were planted as wetland plants into each CW-MFC reactor. Every three days, we collected the effluent overflow of each reactor. At the end of each stage, the wastewater purification performance, electrochemical performance, and physiological and biochemical responses of the *L. hexandra* were analyzed for each CW-MFC system to determine the best HRT of the reactors.

### 3.3. Water Quality Analysis

COD, Cr (VI), total Cr (TCr), ammonia nitrogen (NH_4_^+^-N), total nitrogen (TN), and 4-CP were determined in the effluent water samples. Among them, the water samples for the determination of ammonia nitrogen, Cr (VI) and 4-CP were filtered through a 0.45 -μm PES needle filter. Using the Hach’s COD digestion instrument and COD instrument to measure COD [8]. The TN content was digested by the alkaline potassium persulfate digestion UV spectrophotometric method [87]. The NH_4_^+^-N content was determined by Nessler’s reagent spectrophotometric method [88]. The TCr content was determined by flame atomic absorption spectrophotometry [8]. The Cr (VI) content was determined by diphenylcarbonyl hydrazine spectrophotometry [8]. The 4-CP content was determined by 4-aminoantipyrine spectrophotometry [89].

### 3.4. Electrochemical Performance

The output voltage of each reactor was measured every 8 h using a multimeter and a voltage sensor. By adjusting the resistance value of the resistance box, the potentials of the cathodic and anodic electrodes and the voltages under different external resistances (9000–100 Ω) were determined 15 times by using a multimeter from high to low, each time with a stabilization time of 30 min [8,64]. Currents, current densities, and power densities were calculated in Text S1. Methods to obtain the power density and polarization curve can be found in Text S2.

### 3.5. Physiological and Biochemical Response and Cr Concentration of L. hexandra

At the end of the 15-day experimental stage, we measured the net photosynthetic rate, transpiration rate, stomatal conductance, and intercellular CO_2_ concentration of *L. hexandra* leaves using a photosynthesizer. Subsequently, we harvested *L. hexandra*, cleaned it with deionized water, and divided it into three parts: root, stem, and leaf. Malondialdehyde (MDA) content was determined by the 2-Thiobarbituric acid method, soluble protein content was determined by the Bradford method [90], and chlorophyll content was determined by the ethanol method [91]. The remaining rhizomes and leaves were dried at 105 °C for 30 min and then at 65 °C for 48 h until a constant weight was achieved [8]. The biomass of *L. hexandra* was recorded by weighing the dried plant material using an electronic balance. The chromium content of *L. hexandra* was analyzed by ICP-OES after HNO_3_-H_2_O_2_ wet digestion.

### 3.6. Methods of Analysis

All data were collated using Microsoft 365 Excel, figures were drawn using Origin 2018, and significant differences were analyzed using SPSS 26.0.

### 3.7. Information of Analysis Equipment

The main instruments used during the experiment are shown in Table 5.

## 4. Conclusions

The concomitant contamination of Cr (VI) and 4-CP adversely affects wastewater treatment, electricity generation, plant growth, and chromium enrichment. The implementation of *L. hexandra*-planted CW-MFC systems demonstrates efficacy in removing both 4-CP and Cr (VI). By adjusting the HRT, it was determined that the optimal configuration for wastewater purification and electrochemical performance was achieved at an HRT of 5 d. Among the three CW-MFC reactor configurations tested, CW-MFC-C exhibited the highest wastewater purification efficiency and electricity generation capabilities, outperforming CW-MFC-A and CW-MFC-B. Furthermore, *L. hexandra* exhibited enhanced growth, physiological, and biochemical responses in CW-MFC-C at this HRT. Consequently, an HRT of 5 days was identified as optimal for this system. The significant removal of 4-CP and Cr (VI) at this HRT underscores the efficacy of CW-MFC technology in treating wastewater laden with composite contaminants while generating electricity. However, as previously discussed, numerous factors influence the wastewater treatment and electricity generation performance of CW-MFCs. This study focused solely on the effect of the HRT, but microbial activities and various ecotypes of *L. hexandra* may also play significant roles. Additionally, the power output of CW-MFCs remains suboptimal, and their electricity generation performance requires enhancement. At this stage, CW-MFCs display limited effectiveness as a wastewater treatment process. Although the removal rates of TCr, Cr (VI), and 4-CP from the effluent are high, they still do not reach discharge standards. Consequently, further optimization of the structure and materials is necessary. Thus, CW-MFCs remain in the experimental phase, necessitating extensive research for their advancement towards practical application. Future investigations will assess the influence of microbial activities and different ecotypes of *L. hexandra* to further enhance CW-MFC reactor performance.

## Figures and Tables

**Table 1:** Nomenclature list.

Full Name	Abbreviation
Constructed wetland	CW
Constructed wetland–microbial fuel cell	CW-MFC
Hexavalent chromium	Cr (VI)
p-chlorophenol	4-CP
Electrochemically active bacteria	EABs
Hydraulic retention time	HRT
*Leersia hexandra* Swartz	*L. hexandra*
Chemical oxygen demand	COD
Ammonia nitrogen	NH_4_^+^-N
Total nitrogen	TN
Malondialdehyde	MDA

**Table 3:** Artificial wastewater formulation.

Nutrient Substance	Chemical	Concentration (mg·L^−1^)
Carbon source	CH_3_COONa	192.31
Nitrogen source	NH_4_Cl	107.70
Phosphorus source	KH_2_PO_4_·2H_2_O	19.68
	K_2_HPO_4_	29.16
Trace element	MgSO_4_·7H_2_O	2.46
	FeSO_4_·7H_2_O	0.009
	ZnSO_4_·7H_2_O	0.04
	CuSO_4_·5H_2_O	0.0028
	CaCl_2_	0.0082
	MnCl_2_·4H_2_O	0.008
	CoCl_2_·6H_2_O	0.0029
	NiCl_2_·6H_2_O	0.014
	Na_2_MoO_4_·6H_2_O	0.011

Note: This is a recipe for the 1000 times-concentrated trace element solution.

**Table 4:** Naming conventions for experimental operation periods.

Period	HRT (d)
Stage 1 (S1)	3
Stage 2 (S2)	4
Stage 3 (S3)	5

**Table 5:** List of main experimental instruments.

No.	Instrument Name	Manufacturer
1	UV/Vis Spectrophotometer	Shanghai Metash Instruments Co., Ltd., Shanghai, China
2	Digital multimeter	Shanghai Fluke Test Instruments Co., Ltd., Shanghai, China
3	Peristaltic pump	BaoDing Lead Fluid Technology Co., Ltd., BaoDing, China
4	COD digestion instrument	QingDao Source Enviromental Protection Equipment Co., Ltd., QingDao, China
5	Voltage data sensor	Vernier, Oregon, America
6	ICP-OES	Agilent, California, America
7	Photosynthesizer	Zhe Jiang TOP Instrument Co., Ltd., Hang Zhou, China

## Data Availability

The data that support the findings of this study are available from the corresponding author upon reasonable request.

## Supplementary Materials

The following supporting information can be downloaded at https://www.mdpi.com/article/10.3390/molecules29194773/s1: Figure S1: Cathode and anode stereoscopic structure diagram of different CW-MFC system configurations; Text S1: Formulas to calculate currents, current densities, and power densities; Text S2: Methods to obtained power density and polarization curve [64,92].
