# Supplementary material for: Effects of Hydraulic Retention Time on Removal of Cr (VI) and p-Chlorophenol and Electricity Generation in L. hexandra-Planted Constructed Wetland–Microbial Fuel Cell
Source: Molecules. 2024 Oct 9;29(19):4773. doi: 10.3390/molecules29194773 (PMC11478292; doi:10.3390/molecules29194773)
Supplement: Supplementary file 1 [file molecules-29-04773-s001.zip › molecules-3204495-supplementary.pdf]

Supplementary Material to

Effects of Hydraulic Retention Time on removal of Cr (VI) and p-chlorophenol and electricity generation in *L. hexandra*-planted constructed wetland–microbial fuel cell

Tangming Li [1], Peiwen Yang [1], Jun Yan [1], Mouyixing Chen [1], Shengxiong You [1], Jiahuan Bai [1], Guo Yu [1] , Habib Ullah [2] , Jihuan Chen [1] , Hua Lin [1,3,*]

[1] *College of Environmental Science and Engineering, Guilin University of Technology, Guilin 541000, China*

[2] *Guangxi Collaborative Innovation Center for Water Pollution Control and Water Safety in Karst Areas, Guilin University of Technology, Guilin 541000, China*

[3] *Innovation Center of Yangtze River Delta, Zhejiang University, Zhejiang 311400, China*

* Correspondence: linhua@glut.edu.cn

Summary

Page: 6; Figures: 1; Texts: 2.

- **Figures and Table**

1. **Figure S1.** Cathode and anode stereoscopic structure diagram of different CW-MFC system configurations.

- **Methods:**

1. Text S1. • Formulas to calculate currents, current densities, and power densities.

2. Text S2. • Methods to obtained power density and polarization curve.

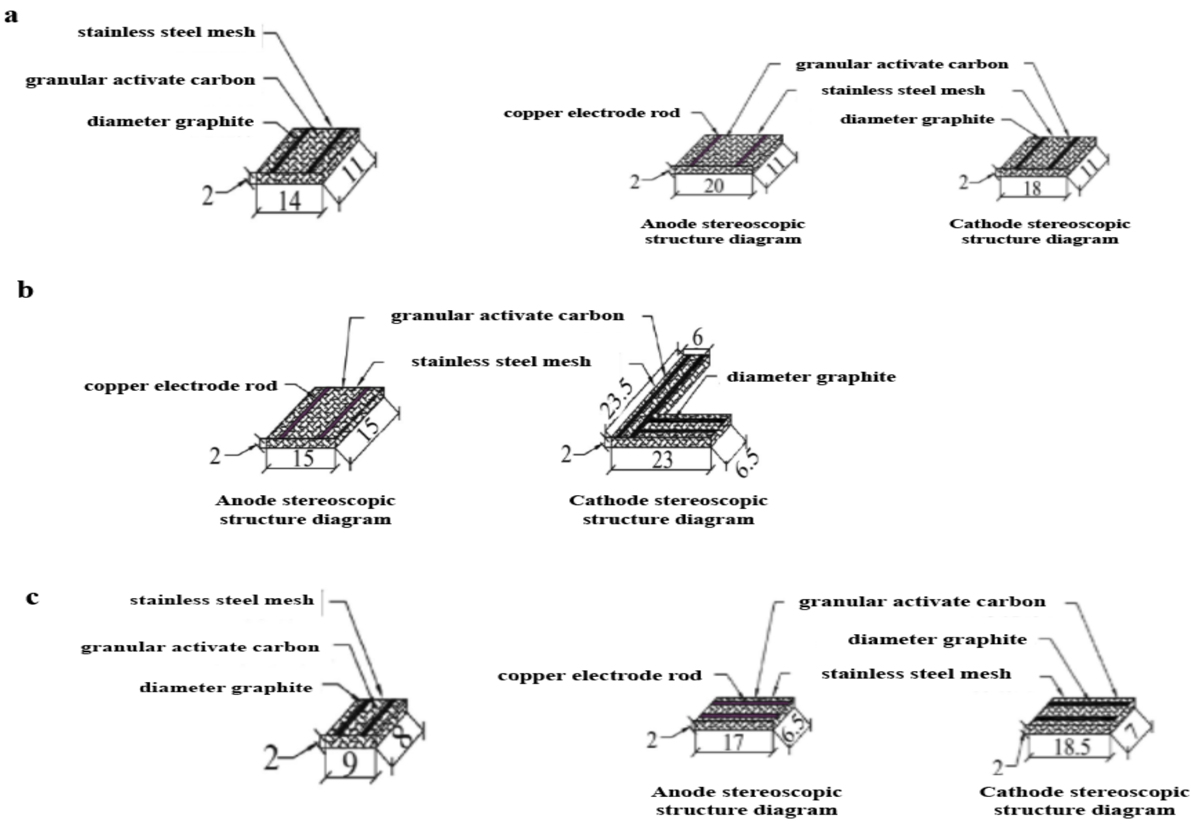

Figure S1. Cathode and anode stereoscopic structure diagram of different CW-MFC system configurations. Note: The units in the figure are cm. (a) Stacked configuration; (b) multistage configuration; (c) modular configuration.

Text S1

- **Formulas to calculate currents, current densities, and power densities [1,2].**

$$I = \frac{U}{R}$$

$$P_V = \frac{U^2}{RV}$$

$$J_V = \frac{I}{V} = \frac{U}{RV}$$

I--- Current, A.

U--- Voltage, V.

R--- External resistances, Ω.

P_V--- Power densities, mW ·m^{-3}.

V--- Anode volume, m^{-3}.

J_V--- Current densities, mA·m^{-3}.

Text S2

- **Methods to obtained power density and polarization curve.**

The power density curve can be obtained by plotting the current density as the horizontal coordinate and the power density as the vertical coordinate, the polarization curve can be obtained by plotting the current density as the horizontal coordinate and the voltage as the vertical coordinate, and the highest point of the power density curve is the highest power density of each system[1,2].

References

1. Sonawane, J.M.; Al-Saadi, S.; Singh Raman, R.K.; Ghosh, P.C.; Adeloju, S.B. Exploring the Use of Polyaniline-Modified Stainless Steel Plates as Low-Cost, High-Performance Anodes for Microbial Fuel Cells. *Electrochimica Acta* **2018**, *268*, 484–493, doi:10.1016/j.electacta.2018.01.163.
2. Wang Y.; Zhang X.; Zheng J.; Zhang Y.; Lin H. Electrochemical properties and microbial community structure of constructed wetland microbial fuel cell under different matrix carbon source. *Chinese Journal of Environmental Engineering* **2021**, *15*, 3696–3706, doi:10.12030/j.cjee.202108060
